# Supplementary material for: Early life stress in male mice blunts responsiveness in a translationally-relevant reward task
Source: Neuropsychopharmacology. 2023 May 31;48(12):1752–9. doi: 10.1038/s41386-023-01610-7 (PMC10579416; doi:10.1038/s41386-023-01610-7)
Supplement: Supplementary file 1 — Supplementary Figures and Legends [file 41386_2023_1610_MOESM1_ESM.docx]

**Supplemental Figure 1**

**
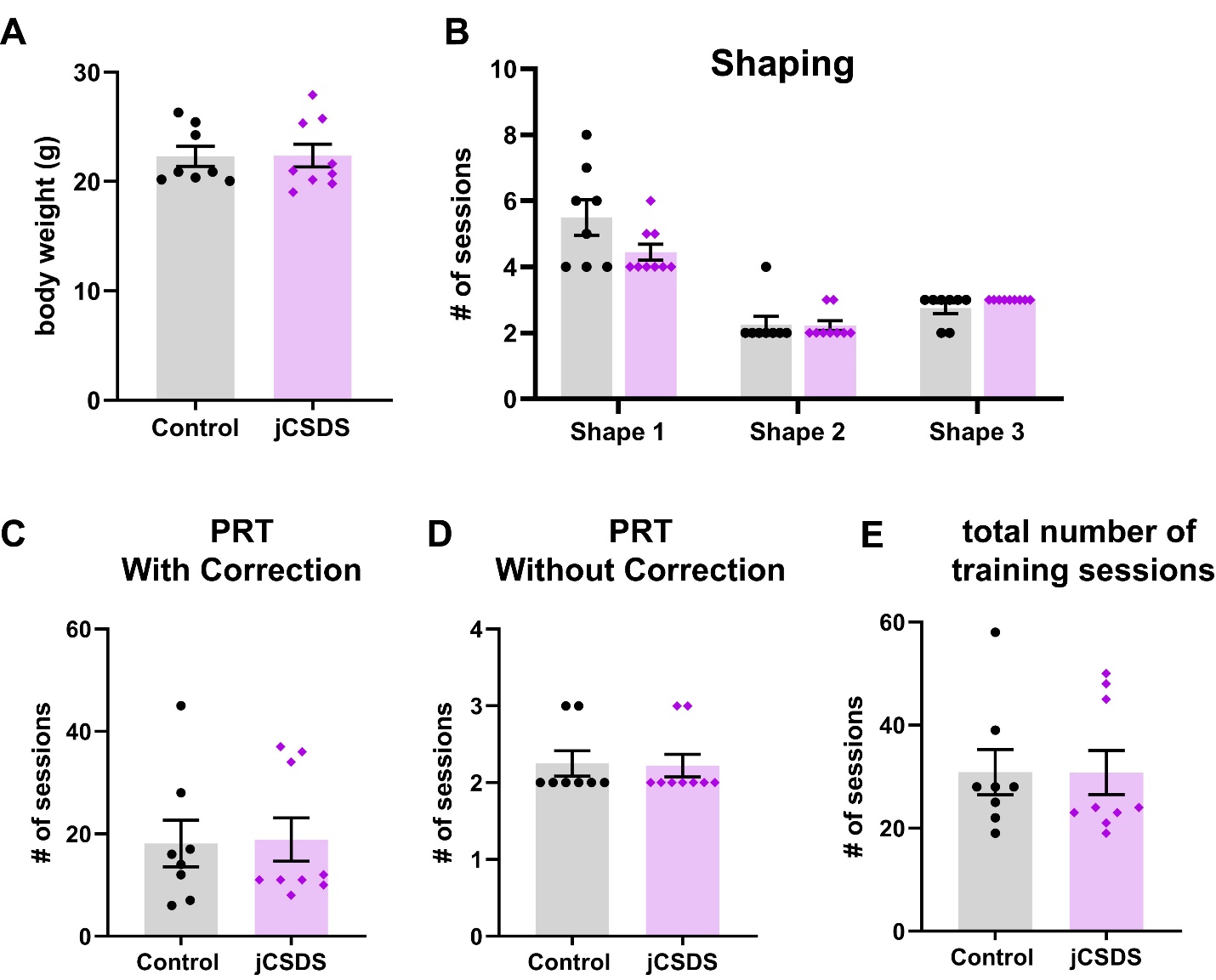
**

**Supplemental Figure 1. Number of sessions to completion for total and individual training phases is similar for control and defeated animals.** A) Average body weight over 5 days of PRT testing. B) Number of sessions to reach criteria for initial shaping (B), corrected (C), and uncorrected (D) phases of PRT training. E) Total number of training sessions to reach PRT testing.

**Supplemental Figure 2**

**
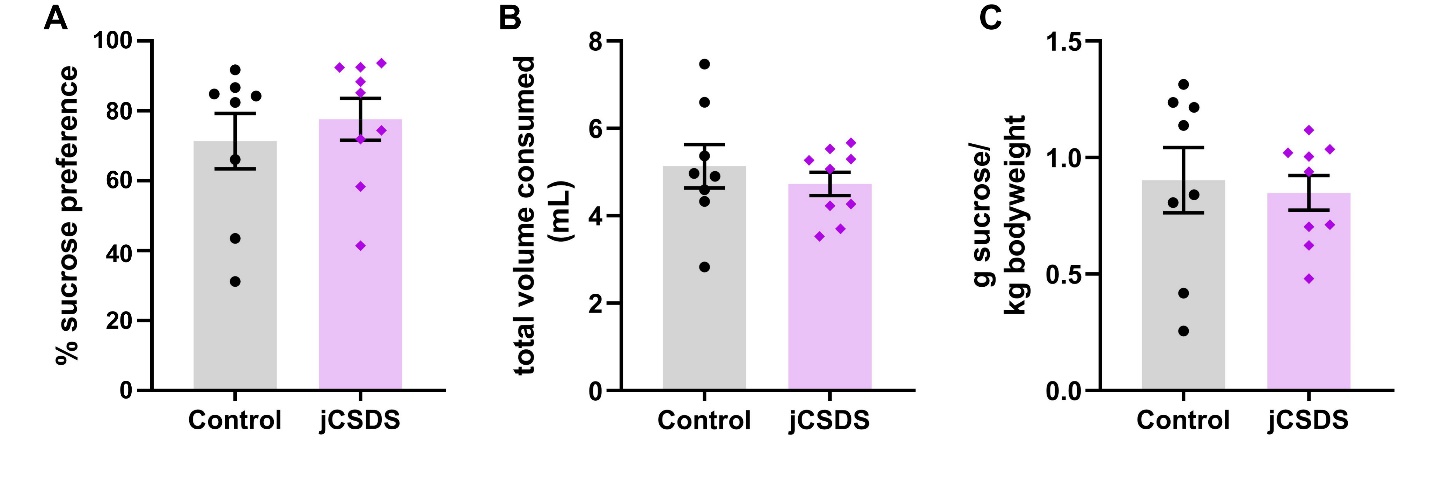
**

**Supplemental Figure 2. Replication of unaffected sucrose preference in adults defeated as juveniles.** Average percent sucrose preference across 3 day testing period (A). Average total volume of sucrose solution and water consumed per day calculated across 3 day testing period (B). Average sucrose consumed per body weight per day calculated across 3 day testing period (C).

**Supplemental Figure 3**

**
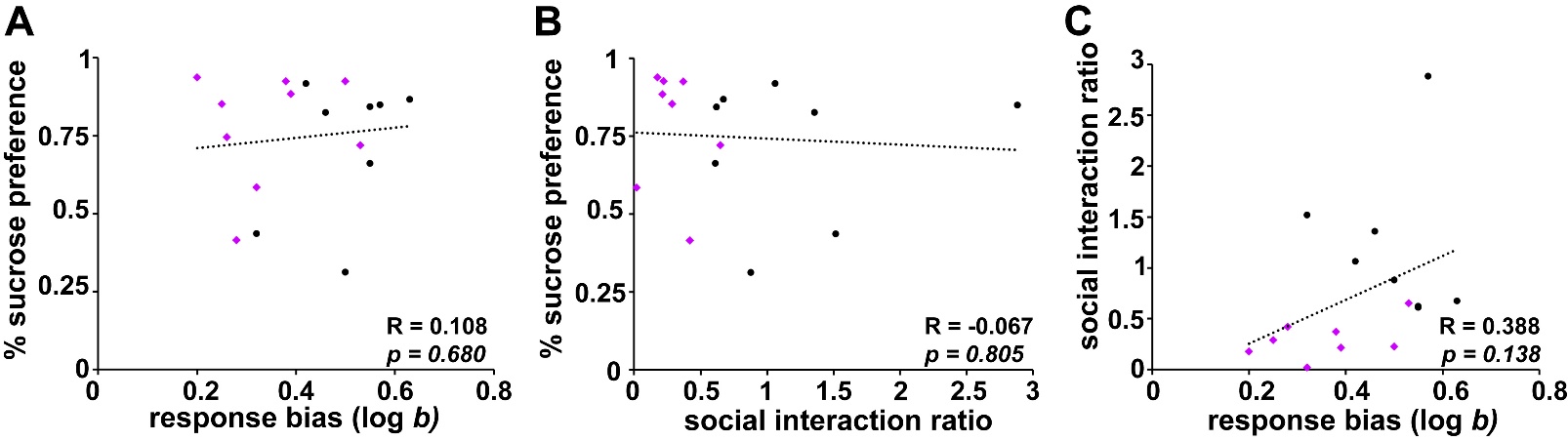
**

**Supplemental Figure 3. Correlations between response bias, sucrose preference, and social interaction.** Correlations between percent sucrose preference as a function of response bias (A) or social interaction ratio (B) as well as social interaction as a function of response bias (C). Dotted lines indicate linear fit to control and defeated data. Pearson correlation coefficient (R) listed for combined control and defeated data.
